# Changes in Social Exclusion Indicators and Psychological Distress Among Homeless People Over a 2.5-Year Period

**DOI:** 10.1007/s11205-016-1486-z

**Published:** 2016-11-15

**Authors:** Barbara Van Straaten, Gerda Rodenburg, Jorien Van der Laan, Sandra N. Boersma, Judith R. L. M. Wolf, Dike Van de Mheen

**Affiliations:** 1 Erasmus Medical Centre, Rotterdam, The Netherlands; 2 IVO Addiction Research Institute, Heemraadssingel 194, 3021 DM Rotterdam, The Netherlands; 3 Impuls - Netherlands Center for Social Care Research, Department of Primary and Community Care, Radboud University Medical Center, Nijmegen, The Netherlands; 4 Amsterdam University of Applied Sciences, Amsterdam, The Netherlands; 5 Department of Health Promotion, Maastricht University, Maastricht, The Netherlands

**Keywords:** Homelessness, Social exclusion, Psychological distress, Cohort study, The Netherlands

## Abstract

Although homelessness is inherently associated with social exclusion, homeless individuals are rarely included in conventional studies on social exclusion. Use of longitudinal survey data from a cohort study on homeless people in four major Dutch cities (*n* = 378) allowed to examine: changes in indicators of social exclusion among homeless people over a 2.5-year period after reporting to the social relief system, and associations between changes in indicators of social exclusion and changes in psychological distress. Multinomial logistic regression analysis was applied to investigate the associations between changes in indicators of social exclusion and changes in psychological distress. Improvements were found in various indicators of social exclusion, whereas financial debts showed no significant improvement. Changes in unmet care needs, health insurance, social support from family and relatedness to others were related to changes in psychological distress. This study demonstrated improvements in various indicators of social exclusion among homeless people over a period of 2.5 years, and sheds light on the concept of social exclusion in relation to homelessness.

## Introduction

Defining homelessness is not as straightforward as it may seem, and the definition of homelessness often varies between countries and/or institutions. The European Typology of Homelessness and Housing Exclusion (ETHOS) provides a broad definition of homelessness and discerns four conceptual categories of homelessness: (1) rooflessness (people living rough, in a night shelter), (2) houselessness (shelter accommodation, supported accommodation for formerly homeless people), (3) insecure housing (temporarily with family/friends, living under threat of violence), and (4) inadequate housing (living in extreme overcrowding, unfit housing) (Amore et al. 2011).

In the Netherlands, a distinction is often made between ‘literal’ homelessness and ‘residential’ homelessness. Literally homeless people are those that do not have their own living accommodation and have no fixed address, sleep rough on the streets, in homeless shelters, in public buildings, or are staying with relatives or friends (Dutch Government and Four Major Cities 2006; Statistics Netherlands 2013; Wolf et al. 2002). Residentially homeless people live in residential homelessness services, such as accommodations for homeless people (e.g. hostels, pensions) (Dutch Government and Four Major Cities 2006; Wolf et al. 2002). In this study, people who reported themselves at the social relief system in the Netherlands were regarded as homeless. Most of them were literally homeless (e.g. sleeping in a night shelter, transitional accommodation or staying temporarily with friends, relatives or acquaintances), a minority were residing in an institution (e.g. a residential care facility) and were residentially homeless, or were housed but were about to be evicted.

Homelessness is inherently associated with social exclusion because the characteristics intertwined with homelessness, such as lack of housing, financial debts and lack of social support (Van Laere et al. 2009; Tsai et al. 2012b; Fazel et al. 2014) are also considered components of social exclusion (Jehoel-Gijsbers and Vrooman 2007; Morgan et al. 2007; Vrooman and Hoff 2013). Homeless individuals can be considered one of the most extreme socially excluded groups in society (European Commission 2009). However, homeless persons are rarely included in conventional studies on social exclusion mainly because they are not a member of a conventional household, which is frequently used as a sample framework in studies on social exclusion (Popay et al. 2008). Therefore, extra attention should be paid to homeless people in research on social exclusion. Therefore, the present study examines changes in social exclusion indicators among homeless people over a 2.5-year period.

### Social Exclusion

Socially excluded persons are persons who experience an accumulation of disadvantages in society (Vrooman and Hoff 2013). Social exclusion is regarded as a multidimensional concept (Sen 2000; Papadopoulos and Tsakloglou 2001; Jehoel-Gijsbers and Vrooman 2007; Poggi 2007; Vrooman and Hoff 2013; Coumans and Schmeets 2015). Although conceptualization of the dimensions which are part of social exclusion varies in the literature on social exclusion, two main dimensions can generally be distinguished: (1) structural-economic exclusion; and (2) socio-cultural exclusion (Vrooman and Hoff 2013). Structural-economic exclusion refers to a distributional dimension and includes a material (income and goods) and a non-material (social rights) aspect. Socio-cultural exclusion refers to a relational dimension and includes social integration which involves: (1) social relations and networks, and (2) cultural integration which concerns values and norms.

Nowadays, the concept of social exclusion is widely applied in the policy context and is a prominent item on the EU’s policy agenda (Papadopoulos and Tsakloglou 2001). It promotes greater coherence between policy domains including economics, education, employment, environment, social affairs and public health (European Commission 2009).

### Social Exclusion and Homelessness

Extreme poverty is regarded as the most important individual predictor of homelessness (Burt 2001).

However, compared to social exclusion, poverty has limited explanatory power with regard to the situation of homeless people. Whereas poverty usually relates to material or economic aspects, social exclusion is a broader concept and provides insight into various aspects of the situation of homeless people; e.g. also including social participation and access to social rights. This concept supports the study of homeless people because this group, in particular, experiences an accumulation of disadvantages. A study among formerly homeless people with severe mental illness reported that social integration can best be treated as a multidimensional construct, including housing as well as factors such as social support (Tsai and Rosenheck 2012). Therefore, the social exclusion construct can be used to examine the situation of homeless people in a holistic sense.

Measuring social exclusion by means of multiple indicators is the most common approach (Morgan et al. 2007). Following Jehoel-Gijsbers et al. (2009), we considered social exclusion as consisting of four dimensions: material deprivation, inadequate access to basic social rights, limited social participation and insufficient cultural integration. The dimension ‘material deprivation’ includes: deficits that people actually experience, as revealed by a lack of basic goods and services for financial reasons, payment arrears, problematic debts, etc. The dimension ‘inadequate access to basic social rights’ means that people do not attain adequate health care, sufficient education and a proper living environment. The dimension ‘limited social participation’ means that people have limited social networks, that they maintain few contacts with others and that their social engagement is low. Finally, the dimension ‘insufficient cultural integration’ refers to a failure to comply with central norms and values of the individual’s community.

These four dimensions, which were developed for the general population, are also relevant for homeless people. For example, concerning the dimension ‘material deprivation’, homeless people often have debts (Van Laere et al. 2009) and have a lack of satisfactory resources for basic needs (Riley et al. 2007). With regard to the dimension ‘access to social rights’, a lack of stable housing is inherently connected to homelessness (Tsemberis et al. 2004), homeless people report substantial unmet care needs (Baggett et al. 2010) and have less health insurance coverage (Kushel et al. 2001). With regard to ‘limited social participation’, having adequate social support is important for the situation of homeless people (Lam and Rosenheck 1999; Thompson et al. 2004; Hawkins and Abrams 2007). For example, prospective studies on stable housing showed that having an intimate partner relationship (Palepu et al. 2010), having others who are dependent on the homeless person for food/shelter (Orwin et al. 2005) and adequate family support (Caton et al. 2005) were positive predictors of attaining stable housing after a period of homelessness. However, being homeless makes it more difficult to connect to people who are not homeless. Finally, with regard to ‘insufficient cultural integration’, homeless people are substantially more involved in the criminal justice system (McGuire and Rosenheck 2004).

However, the operationalization of these four dimensions in the index of Jehoel-Gijsbers et al. (2009) is aimed at the general population and includes indicators such as ‘I have contact with my neighbors’ (dimension of social participation) and ‘I have enough money to heat my home’ (dimension of material deprivation). Clearly, these indicators are not appropriate for homeless people. Therefore, we explored social exclusion related to the four dimensions of social exclusion of Jehoel-Gijsbers et al. (2009), but adapted them to the situation of homeless people. We used at least two indicators for each of the four dimensions of social exclusion. The indicators we explored in this study are:

- material deprivation: debts (Van Laere et al. 2009) and lack of satisfactory resources for basic needs (Riley et al. 2007);
- inadequate access to social rights: lack of stable housing (Tsemberis et al. 2004), unmet care needs (Baggett et al. 2010) and less health insurance coverage (Kushel et al. 2001);
- limited social participation: social support (i.e. social support from family/friends, and relatedness to others) (Lam and Rosenheck 1999; Thompson et al. 2004; Hawkins and Abrams 2007) and employment (i.e. paid or voluntary work) (Zuvekas and Hill 2000);
- insufficient cultural integration: involvement in the criminal justice system (i.e. being arrested and receiving fines) (McGuire and Rosenheck 2004).

By using these indicators, we selected those indicators empirically shown to be important when studying homeless people, while keeping in mind the four-dimensional concept of social exclusion developed for the general population.

### Social Exclusion and Psychological Distress

Among the homeless, (mental) health problems are often present (Schanzer et al. 2007; Fazel et al. 2008; Nielsen et al. 2011; Nusselder et al. 2013; Krausz et al. 2013; Fazel et al. 2014; Toro et al. 2014). Among the general population, health is strongly related to social exclusion (Santana 2002; Popay et al. 2008; Coumans and Schmeets 2015; Evans-Lacko et al. 2014); e.g. those who are socially excluded generally have a significantly poorer mental health than the non-excluded (Payne 2006; Jehoel-Gijsbers and Vrooman 2007). Apart from a relationship between mental health and social exclusion in general, mental health is also related to separate indicators of social exclusion. For example, relationships have been demonstrated between debts and mental health (Richardson et al. 2013), between social support and mental health (Kawachi 2001; Tsai et al. 2012a) and between employment and mental health (Thomas et al. 2005). However, to our knowledge, these relationships have not been investigated among homeless persons, which makes it highly relevant to investigate the relationship between mental health and indicators of social exclusion among homeless people. Also, because the profile of the homeless population (in the Netherlands) has changed substantially over recent years, including more homeless families (Planije and Tuynman 2015), it has practical relevance for policy makers, social relief facilities and aid agencies to gain insight into processes of social exclusion and psychological distress.

We examined mental health using the concept of psychological distress. In both research and clinical settings, psychological distress is a widespread indicator of mental health and mainly combines depression and anxiety symptoms that are indicative of feelings of emotional ill-being (Drapeau et al. 2010). Examining various social exclusion indicators in one model allows us to elucidate which indicators of social exclusion have the strongest association with mental health among homeless people. We also explored whether changes in indicators of social exclusion over time are associated with changes in mental health. Change scores for all the indicators in this study (i.e. debts, resources for basic needs, stable housing, unmet care needs, health insurance, social support from family, social support from friends, relatedness to other, work or voluntary work, arrests and received fines) were included in a model to investigate the associations with changes in mental health among the participants.

### Study Aim and Hypotheses

This study aimed to examine changes in indicators of social exclusion in a cohort of Dutch homeless people over a period of 2.5 years using a four-dimensional concept of social exclusion (Jehoel-Gijsbers et al. 2009). This study also addressed associations of changes in indicators of social exclusion with changes in psychological distress over a period of 2.5 years (we did not aim to study causal relationships). Using longitudinal data of a cohort study among Dutch homeless people (*n* = 378) enabled us to report on changes in indicators of social exclusion and changes in psychological distress over a period of 2.5 years after the homeless people had entered the social relief system (see Methods for details on the study population).

Because people who are more socially excluded generally have a poorer mental health than the non-excluded (Payne 2006; Jehoel-Gijsbers and Vrooman 2007) and a relationship has been shown between several social exclusion indicators (e.g. debts, social support, employment) and mental health (Kawachi 2001; Thomas et al. 2005; Tsai et al. 2012a; Richardson et al. 2013), we hypothesized that improvements on indicators of social exclusion (e.g. having more social support, having less high debts) among homeless people would be associated with improvements in psychological distress (i.e. reduced psychological distress).

## Methods

This study used longitudinal survey data from a cohort study (CODA) on homeless people in the four major cities of the Netherlands (Amsterdam, The Hague, Rotterdam and Utrecht, together called ‘the G4’). CODA-G4 is a multi-site cohort study following homeless persons for a period of 2.5 years, starting from the moment they reported themselves at a central access point for social relief in 2011 in one of the included cities and were accepted for an individual program plan within the CODA-G4 study. The main objectives of CODA-G4 were to determine among homeless individuals accepted for an individual program plan: their care needs and goals in relation to their background and problems, housing transitions and predictors of stable housing, and changes in their living situation (including health, work/finances, social relations, criminal activities), and quality of life as well as predictors of quality of life. The study did not aim to investigate the impact of policy measures (e.g. the individual program plan) on the living situation of the participants.

In the Netherlands it is obligatory for every homeless person to report at a central access point to get access to social relief facilities, such as a night shelter. The delivery of care and the supply of living accommodation after accepting an individual program plan is provided by local care agencies. The municipalities act as policy co-ordinators and case managers monitor the execution of the individual program plan. In 2011, over 1800 adults and 1100 young adults reported themselves at a central access point for social relief and were accepted to start an individual program plan in the four major cities of the Netherlands (Tuynman and Planije 2012). This implies that they satisfied the criteria set by the four major Dutch cities at that time for starting an individual program plan, i.e. being at least 18 years of age, having legal residence in the Netherlands, residing in the region of application for at least 2 years during the last 3 years, having abandoned the home situation, and being unable to hold one’s own in society. All these persons were potential participants for this study. Consequently, other subgroups (such as undocumented homeless people) were not provided with an individual program plan and were therefore excluded from this study.

### Data Collection

At the start of the study in January 2011, the potential participants were approached either at a central access point for social relief (one in each city) by an employee of the access point, or at temporary accommodation (where they stayed shortly after entering the social relief system) by the researchers or interviewers. When a potential participant expressed interest in taking part in the study, the researchers contacted that person to explain the study aims, the interview procedure, and the informed consent procedure. When the participant agreed to participate, an interview appointment was scheduled. In total, 513 participants agreed to participate: 410 homeless adults (aged ≥23 years) and 103 young adults (aged 18–22 years). The number of participants in each of the four cities was in accordance with the inflow of homeless people at the central access points for social relief in the particular city. No data were available on how many potential participants were approached and how many refused to participate. Therefore, in order to obtain information about the representativeness of the study participants, we compared the total group of homeless adults and youth who reported themselves at a central access point for social relief in one of the four included cities in 2011, with our study participants. Adult participants (aged ≥23 years; *n* = 410) were representative in terms of age and gender. Youth participants (aged 18–22 years; *n* = 103) were representative in terms of age but, in our sample, males were overrepresented (60.2% younger males in the cohort vs. 49.2% younger males in the total group).

#### Follow-Up Measurements

Participants were contacted at 6, 18 and 30 months after the first measurement by telephone, e-mail, letter, their social network (family, friends and care providers), or private messages via social media. Participants who were lost to follow-up at one or more measurement were again contacted for the next measurement(s).

**Fig. 1:**
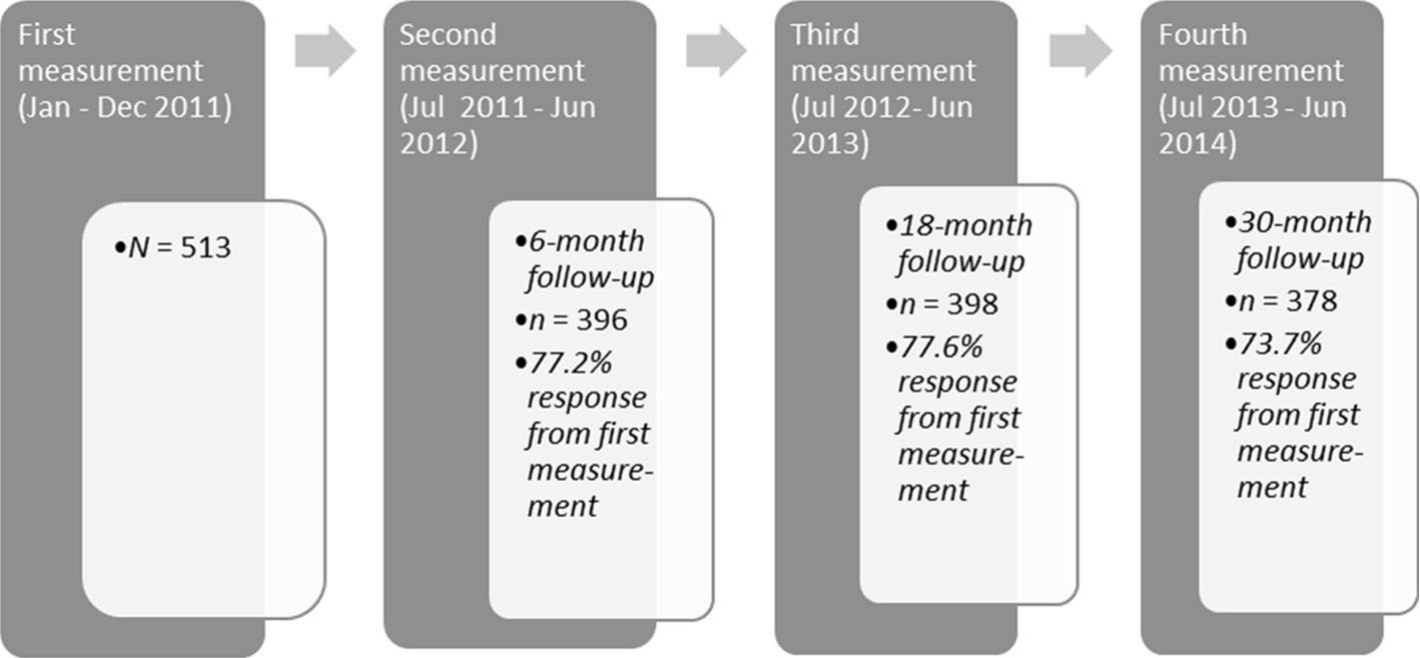
Sample size and response per measurement during the study

Data for this study were derived from the baseline interview which took place shortly after the participants reported themselves at a central access point for social relief (T0; January 2011–December 2011) and from the fourth interview which took place 2.5 years after the baseline interview (T3; July 2013–June 2014). Of the initial cohort of 513 participants, 378 participants (73.7%) completed the fourth interview [see Fig. 1 for overall sample size and response per measurement during the study; (for detailed information on number of participants and key characteristics per city, see Van Straaten et al. 2013a, b, c, d)]. Although we do not have information about the reasons for attrition of all the 135 non-respondents, we know that some no longer wished to participate in the study and that one participant had died. To investigate selective loss to follow-up, we compared respondents on the final interview (*n* = 378) with non-respondents (*n* = 135) on demographic variables (age, gender, education, ethnicity) as reported at the first measurement. Compared to respondents on the final interview, non-respondents were on average younger (33.8 vs. 37.2 years), were more often male (83.0 vs. 74.3%) and more often had the lowest level of education (44.4 vs. 30.2%). No selective loss to follow-up was found with respect to ethnicity.

### Measures

#### Demographic Characteristics

Demographic characteristics including gender, age, ethnicity and educational level were assessed. Ethnicity was categorized into ‘native Dutch’ when the participant and both parents were born in the Netherlands and as ‘non-native Dutch’ when participants were foreign born or when participants were born in the Netherlands but one or both of their parents were foreign born. Education was categorized as ‘lowest’ when the participant completed primary education at the most, as ‘low’ when the participant completed pre‐vocational education, lower technical education, assistant training or basic labor-oriented education, as ‘intermediate’ when the participant completed secondary vocational education, senior general secondary education or pre-university education, and categorized as ‘high’ when the participant completed higher professional education or university education.

#### Debts

The amount of debts (not including mortgages without overdue payments) was assessed: debts reported by participants showed a very skewed distribution with various outliers (range of the continuous data: 0–500,000 euros). Therefore, we dichotomized debts into ‘1000 euros or more’ (high; >first quartile) and ‘less than 1000 euros’ (low; <first quartile). This cut-off between high and low debts was data-driven because no normative data for the amount of debts were available.

#### Resources for Basic Needs

The Dutch abbreviated version of the Lehman Quality of Life Interview (Wolf et al. 2002) was used to assess the adequacy of finances to cover basic expenditures. Participants were asked “During the past month, did you generally have enough money to cover (1) food, (2) clothing, (3) housing, (4) traveling around the city for things like shopping, medical appointments, or visiting friends and relatives, and (5) social activities like movies or eating in restaurants?” (yes or no). The mean number of covered expenditures (range 0–5) was calculated.

#### Stable Housing

Stable housing was defined as at least 90 consecutive days independently housed or living in supportive housing (owned by care organizations) (yes or no) (Van Straaten et al. 2016).

#### Unmet Care Needs

Unmet care needs were assessed using a questionnaire developed by Impuls - Netherlands Center for Social Care Research (Lako et al. 2013). The response categories were based on the format of the Short-Form Quality of Life and Care questionnaire (QoLC) (Wennink and Wijngaarden 2004). Care needs were considered on eight life domains: finding housing, finances, basic skills, searching for work, physical health, mental health, dental care and safety. For each domain, two questions were asked: “Do you want help with …?” and “Do you get help with … ?”. An unmet care need variable was created for each life domain, which is scored affirmatively when participants indicated they wanted help, but did not receive help. All unmet care needs were summed to a total unmet needs variable, ranging from 0 to 8. The questionnaire has previously been used among homeless youth (Krabbenborg et al. 2013) and abused women (Jonker et al. 2012).

#### Health Insurance

We asked participants: “Do you have health insurance?” (yes or no).

#### Social Support from Family and from Friends

Social support was assessed by five items derived from scales developed for the Medical Outcome Study (MOS) Social Support (Sherbourne and Stewart 1991). Participants were asked to indicate how often different kinds of support were available to them through family and friends or other acquaintances, on a 5-point scale ranging from ‘none of the time’ to ‘all of the time’. Two social support measures (ranging from 0 to 5) were constructed by averaging across items: a family measure, and a friends and acquaintances measure. The MOS Social Support Survey has been used in several studies among homeless people (O’Toole et al. 1999; Nyamathi et al. 2000) and showed high convergent and discriminant validity and internal consistency (Sherbourne and Stewart 1991). The selection of items used in the present study has been successfully used in previous longitudinal research among homeless populations (Lako et al. 2013; Krabbenborg et al. 2013).

#### Relatedness to Others

Experiences of relatedness were measured by one subscale of the Basic Psychological Needs questionnaire, based on the basic psychological need satisfaction-work version (Ilardi et al. 2006). Participants were asked to indicate their agreement with 7 items on a 7-point Likert scale, ranging from not true at all (1) to definitely true (7). An example of an item is: ‘People in my life care about me’. The scale has been used in previous studies (Gagné 2003), including a study among homeless youth (Krabbenborg et al. 2013). Adequate factor structure, internal consistency, reliability (Cronbach’s alpha = .92), discriminant validity and predictive validity have been demonstrated (Vlachopoulos and Michailidou 2006; Johnston and Finney 2010). The relatedness subscale score ranges from 0 to 7 and was constructed by averaging across the items of the subscale.

#### Having a Job/Volunteer Work

The Dutch abbreviated version of the Lehman QoL Interview (Wolf et al. 2002) was used to assess whether participants had a job by asking: “Do you have a job at this moment (paid job or volunteer work)?” (yes or no).

#### Arrests and Fines

The Dutch abbreviated version of the Lehman QoL Interview (Wolf et al. 2002) was used to assess whether participants had been arrested by asking: “Have you been arrested or picked-up for any crimes in the past year?” (yes or no), and “Did you get any fines for any violations of the law in the past year?” (yes or no).

#### Psychological Distress

The Brief Symptom Inventory 18 (BSI-18) was used to measure psychological distress (Derogatis 2001). The BSI-18 is a short form consisting of 18 items taken from the Symptom Checklist-90-R (SCL-90-R) (Derogatis 1994), which correlates highly with the SCL-90-R. The BSI-18 assesses three symptom scales (i.e. depression, anxiety and somatization), which are included in a total score as an indication of general psychological distress. The BSI is a frequently used measure to evaluate psychological distress in studies among homeless populations (McCaskill et al. 1998; Kashner et al. 2002; Ball et al. 2005; Weinreb et al. 2006; Tsemberis et al. 2012). Respondents rated, from 0 (*never experience symptom*) to 4 (*very often experience symptom*), 18 items like “Nervousness or shakiness inside” and “Feelings of worthlessness”. The Dutch translation was used, with (provisional) norm scores for the Dutch population (De Beurs 2011). We compared the scores of the participants with the norm scores described in the manual for the Dutch community sample, with separate norm scores for men and women, and for different age categories (18–29 years and 30+ years) (De Beurs 2011). Because norms for t-scores are not available for the Dutch BSI-18 (De Beurs 2011), participants were categorized as having a high level of psychological distress if they scored in the upper 20^th^ percentile on a subscale compared with a Dutch community sample. Participants were categorized into two groups: participants with a high level and participants with less than a high level of psychological distress.

#### Change Variables

To create a change variable for psychological distress, participants were classified as having “reduced psychological distress” when they had a high level of distress at baseline and no high level of distress at follow-up, as “increased psychological distress” when they had no high level of distress at baseline, but a high level of distress at follow-up, and as “no change in psychological distress” when they had both at baseline and follow-up either a high or no high level of psychological distress. For all other variables, a change variable was created by subtracting the score at baseline from the score at follow-up.

### Statistical Analysis

Descriptive analyses were performed to describe demographic characteristics of the participants.

To analyse changes in indicators of social exclusion between the baseline measurement and the 2.5-year follow-up, a McNemar test was used for 2 × 2 categorical data. A paired *t* test was used to analyse changes between baseline measurement and the 2.5-year follow-up for the continuous data.

We used a multinomial logistic regression, adjusted for age and gender, to analyze the association between the change in social exclusion indicators between baseline and follow-up and the change in psychological distress between baseline and follow-up. This type of regression is similar to binary logistic regression, but allows the dependent variable to have more than two categories. In this study the outcome variable ‘change in psychological distress’ consisted of three categories: ‘decreased psychological distress’, ‘increased psychological distress’ and ‘no change in psychological distress’. The reference (or excluded) category for this analysis was ‘no change in psychological distress’. Participants with missing data were excluded from the analyses.

Results are reported as odds ratio (OR), standard error (SE) of the OR, and the *p* values. The Nagelkerke R^2^ is reported to indicate the proportion of variance of the change in psychological distress that was explained by all the indicators in the model. We used partial Nagelkerke’s R^2^ to quantify the partial contributions of each indicator to the change in psychological distress. Multicollinearity (i.e. when two or more variables are very closely linearly related) among the predictors was examined by (a) the variance inflation factor (VIF) (indicated by a VIF value >10) and by (b) the tolerance value (indicated by a value <0.1); the Nagelkerke R^2^ is reported. All statistical analyses were conducted with IBM SPSS statistics version 23.

## Results

### Descriptive Statistics

Table 1 presents the demographic characteristics of the participants at baseline. The mean age of the participants was 37.2 (range 18–71) years. Almost three quarters were male (74.3%), and the majority had a non-native Dutch background (64.1%); 30% fell in the lowest category of education (completed primary education at the most), and 45.2% were had a low level of education.

**Table 1:** Demographic characteristics of the participants in this study (n_range_ = 368–378) and the total study population (n_range_ = 498–513)

Baseline characteristic	Participants		Total study population	
	n	%/mean (SD)	n	%/mean (SD)
Age in years	378	37.2 (12.9) (range 18–71)	513	36.3 (13.0) (range 18–71)
Gender %	378		513	
Male		74.3		76.6
Female		25.7		23.4
Education %	374		509	
Lowest		30.2		34.0
Low		45.2		42.2
Intermediate		15.8		15.9
High		8.8		7.9
Ethnicity %	368		498	
Native Dutch		35.9		35.7
Non-native Dutch		64.1		64.3

### Changes in Social Exclusion Indicators and Changes in Psychological Distress

Table 2 shows the changes in the social exclusion indicators and in psychological distress during the period between entering the social relief system (T0) and 2.5 years later (T3). Significant improvements took place on most of the social exclusion indicators in the 2.5 years after admission to the social relief system. The percentage of stably housed participants rose sharply by 66.7% (for more findings on housing stability and on predictors of housing stability, see Van Straaten et al. 2016). Also, most of the ‘social rights’ indicators improved significantly, as did the indicators conceptualizing social participation and cultural integration. The only indicator that did not improve significantly was ‘high debts’: i.e. the percentage of participants with high debts (≥1000 euros) remained at around 71%.

**Table 2:** Changes in social exclusion indicators and psychological distress in the period between entering the social relief system (T0) and 2.5 years later (T3) among the initially homeless participants

Social exclusion dimensions	Social exclusion indicators	Range of scores/coding	*n*	T0 (% or *M)*	T3v(% or *M*)	Change T3 − T0 (p.p. or *M*)
Material deprivation	High debts (≥1000 euros)	Yes = 1; no = 0	280	71.4%	70.7%	−0.007
	Satisfied resources for basic needs	0–5	376	2.11	2.87	0.76***
Access to social rights	Stable housing	Yes = 1; no = 0	378	0.0%	66.7%	0.667^n.a.^
	Number of unmet care needs	0–8	376	2.19	1.26	−0.93***
	Health insurance	Yes = 1; no = 0	367	91.3%	96.2%	0.049**
Social participation	Social support from family^a^	0–5	366	2.82	3.62	0.80***
	Social support from friends^b^	0–5	378	3.11	3.60	0.48***
	Relatedness to others^c^	0–7	369	4.97	5.19	0.22***
	Work or voluntary work	Yes = 1; no = 0	378	30.4%	38.1%	0.077*
Cultural integration	Arrested in the past year	Yes = 1; no = 0	371	31.3%	8.6%	−0.227***
	Received fines in the past year	Yes = 1; no = 0	372	47.8%	29.0%	−0.188***
Psychological distress						
Psychological distress	High level of psychological distress	Yes = 1; no = 0	367	39.5%	27.0%	−0.125***

*p.p.* percentage points, *n.a.* significance testing not possible due to small cell counts

* *p* < .05; ** *p* < .01; *** *p* < .001

^a^Higher scores indicating greater social support from family

^b^Higher scores indicating greater social support from friends

^c^Higher scores indicating more feelings of relatedness

There was a significant decrease in the number of participants with a high level of psychological distress: i.e. at baseline 39.5% of the participants had a high level of psychological distress compared with 27.0% 2.5 years later.

### Associations Between Changes in Social Exclusion Indicators and Changes in Psychological Distress

Of the participants (*n* = 367), 19.3% (*n* = 71) showed a substantial decrease in psychological distress: i.e. they had a high level of psychological distress at baseline but no high level of psychological distress was present at 2.5-year follow-up. An increase in psychological distress was seen in a relatively small group of participants (6.8%, *n* = 25), while for most participants their distress level had not changed (73.8%, *n* = 271).

Multinomial logistic regression analysis, adjusted for age and gender, revealed reduced psychological distress more frequently in participants reporting less unmet care needs at 2.5-year follow-up (OR 0.75), less health insurance coverage (OR 0.26), more social support from family (OR 1.58) and more feelings of relatedness (OR 1.82) (Table 3).

**Table 3:** Associations between changes in social exclusion indicators and changes in psychological distress among the initially homeless participants (n = 246)

Changes in social exclusion indicators^a^	Reduced psychological distress (*n* = 49)	Increased psychological distress (*n* = 22)
	OR^b,c^ (95 % CI)	OR^b,c^ (95 % CI)
Material deprivation		
Change in high debts	0.844 (0.40–1.78)	1.02 (0.31–3.34)
Change in satisfied resources for basic needs	1.06 (0.87–1.30)	1.04 (0.78–1.39)
Access to social rights		
Change in stable housing	1.02 (0.46–2.26)	1.22 (0.37–3.98)
Change in unmet care needs	0.75 (0.59–0.95)*	1.92 (1.25–2.95)**
Change in health insurance	0.26 (0.071–0.95)*	7.86 (1.29–47.9)*
Social participation		
Change in social support from family	1.58 (1.18–2.12)**	0.81 (0.53–1.24)
Change in social support from friends	0.99 (0.73–1.35)	1.29 (0.81–2.04)
Change in relatedness to others	1.82 (1.11–2.98)*	0.28 (0.12–0.68)**
Change in work or voluntary work	1.37 (0.72–2.61)	0.52 (0.18–1.47)
Cultural integration		
Change in arrests in the past year	0.75 (0.34–1.62)	3.26 (0.97–11.0)
Change in received fines in the past year	1.24 (0.70–2.21)	1.67 (0.64–4.38)

Nagelkerke R^2^ = 0.371; All the VIF values for the predictors were <10 and all the tolerance values were >0.1, indicating that there was no multicollinearity in the model

* *p* < .05; ** *p* < .01; *** *p* < .001

^a^Change scores of the indicators are used in this model (score at baseline subtracted from the score at follow-up)

^b^Adjusted for all other variables included in the multivariate model and for age and gender

^c^‘No change in psychological distress’ (*n* = 175) was the reference category

Increased psychological distress was found more frequently in participants reporting more unmet care needs (OR 1.92), more health insurance coverage (OR 7.86) and less feelings of relatedness (OR 0.28) (Table 3).

These variables together accounted for 37.1% of the variance in the change in psychological distress between baseline and 2.5 year follow-up. The four variables that contributed most to this rate (partial Nagelkerke’s R^2^) were: change in unmet care needs (12.4%), change in relatedness to others (8.6%), change in social support from family (4.0%), and change in arrests in the past year (3.5%).

Because 98 participants could not provide data on debts on either the first measurement, the follow-up measurement or on both measurements (they did not know the extent of their debts), they were excluded from the model. Additional analysis revealed that excluding these participants had no significant impact on the results (not displayed).

## Discussion

This study examined changes in indicators of social exclusion in a cohort of Dutch homeless people over a period of 2.5 years using a four-dimensional concept of social exclusion (Jehoel-Gijsbers et al. 2009). This study also addressed associations of changes in indicators of social exclusion with changes in psychological distress.

### Changes in Indicators of Social Exclusion Over 2.5 Years

For all of the four dimensions of social exclusion, i.e. ‘material deprivation’, ‘access to social rights’, ‘social participation’ and ‘cultural integration’, at least one indicator improved significantly between baseline and 2.5-year follow-up. With regard to the dimension ‘material deprivation’, participants reported more satisfied resources for basic needs (e.g. having enough money to cover food, clothing, housing, etc.). However, financial debts did not significantly improve and the majority of the participants still had high debts at follow-up. With regard to ‘access to social rights’, the results showed a remarkable improvement in the indicator ‘stable housing’: when entering the social relief system none of the participants were stably housed whereas 2.5 years later 66.7% were stably housed. Also, less unmet care needs and more health insurance coverage were reported. With regard to ‘social participation’, there was more social support from family and friends, more relatedness to others, and more participants had a job or voluntary work. Finally, with regard to ‘cultural integration’, less participants had been arrested or received fines.

### Associations Between Changes in Indicators of Social Exclusion and Changes in Psychological Distress

This study identified changes in social exclusion measured by means of various indicators which were associated with changes in psychological distress: participants reporting less unmet care needs, less health insurance coverage, more social support from family and more feelings of relatedness reported reduced psychological distress more frequently. Conversely, an increase in unmet care needs, relatedness to others and more health insurance coverage were related to an increase in psychological distress. The predictor variables together accounted for 37.1% of the variance in the change in psychological distress between baseline and 2.5 year follow-up.

The association between social exclusion indicators and psychological distress is in line with results from the general population, where it was found that those who are socially excluded generally have a significantly poorer mental health than the non-excluded (Jehoel-Gijsbers and Vrooman 2007). In addition, a study among clients of mental health centers found that those who had more severe mental health symptoms were less socially integrated, i.e., they reported less relationship contacts and less social support (Tsai et al. 2012a). Our study adds information on this relationship, because this has not previously been investigated specifically for homeless people in a longitudinal way. However, we cannot make causal relationships based on our data. The relationship between social exclusion and mental health is complex: i.e. many of the elements of social exclusion (e.g. low income, lack of social networks, not having a job) could (in different circumstances) be both causal factors and consequences of mental health problems (Sayce 2001).

It is noteworthy that the indicator ‘stable housing’ was not associated with reduced psychological distress. This suggests that housing in itself is not sufficient to improve homeless people’s mental health. A previous study showed that housing homeless people does not automatically lead to social integration (Tsai et al. 2012b); this latter study among homeless adults after entering a supported housing program, found that although the improvement in housing was substantial, changes in other domains of social integration were minimal. The authors concluded that clients may benefit from interventions that focus on their social integration only after housing is obtained. Our results indicate that such an intervention could be beneficial for improving the mental health of homeless people regardless of their housing situation.. The beneficial effects of social support for the mental health of persons with mental illness have previously been demonstrated (Albert et al. 1998; Hawkins and Abrams 2007).

The finding that less unmet care needs were associated with reduced psychological distress may have various explanations: for example, the participants may have had less unmet care needs because of the improvements that took place in their living situation, or the unmet care needs may have been addressed within the 2.5-year study period, resulting in an improvement in mental health.

An unexpected result was that the coverage of health insurance was negatively associated with reduced psychological distress. However, although this result was significant, the practical relevance of this finding is unclear. In the Netherlands, it is mandatory for all residents to take out health insurance, and every health insurer in the Netherlands has a legal obligation to accept everyone who applies for insurance. Uninsured persons are identified by means of database comparisons and, if they refuse to comply to take out insurance, the Health Insurance Board will take out insurance on behalf of anyone who is still uninsured (The Ministry of Health Welfare and Sport 2011). Therefore, the prevalence of people who are uninsured in the Netherlands is very low; this also applies to homeless people who legally reside in the Netherlands (e.g. in our cohort, ≥90% had health insurance). However, it is possible that health insurance coverage was the most prevalent among individuals with the most serious (mental) health problems, because they may be in more contact with care providers who take out insurance for their clients; this might be an explanation for this particular result.

### No Improvements in ‘High Debts’

Despite the fact that the policy measures for homeless people were also aimed to improve income and target debts, having high debts was the only indicator of social exclusion that did not improve. In the Netherlands, there is an arrangement for debtors who are not able to pay their creditors. In this process the debtor becomes debt free after a 3-year period whereby the creditors receive a portion of the money owed to them. A possible explanation for the finding that high debts among the participants did not decline, is that it may take more time for an intervention to have its effect on debts. A longer follow-up period might show a decline in debts in the subgroup with this arrangement for debtors. High debts are also associated with poorer health outcomes (Clayton et al. 2015). However, we found no association between a change in debt and a decrease in psychological distress. The absence of this association might be explained by the small number of persons who reported an improvement in debts between baseline and follow-up. Additional analyses revealed a significant association between high debts and a high level of psychological distress at baseline as well as at follow-up. Thus, if reductions in high debts take place in future among these participants, this indicator of social exclusion might also be significantly associated with reduced psychological distress.

### Newly Homeless People

Our participants consisted mainly of ‘newly homeless people’; i.e. those who reported to the social relief system in 2011. More than half of them had a total duration of homelessness in their lives of ≤1 year (Van Straaten et al. 2012). Investigating a cohort of mainly ‘newly homeless people’ in terms of social exclusion is very relevant. First-time homeless people often return to independent housing, but remain a vulnerable group after exiting the shelter and returning to the poor communities from which they often emerged (Caton et al. 2005). This relatively short duration of homelessness seems to fit a trend in the Netherlands towards a shorter mean duration of homelessness among homeless people. To illustrate, from around the turn of the century until about 10 years ago (2001–2006), studies among Dutch homeless people showed that the mean duration of homelessness was around 6 years (Reinking et al. 2001; De Bruin et al. 2003; Hulsbosch et al. 2005; Vocks et al. 2007). Being homeless can then gradually develop into a way of life: they socialise with other homeless people, they are seen as homeless by the environment, and may start viewing themselves as such (Van Doorn 2002). More recent studies report substantially shorter mean durations of homelessness of around 3 years (Tielen 2010) to as short a duration as a few months (Van Everdingen 2015). Although these variations in the duration of homelessness might be influenced by the type of facility in which a study is conducted, this trend suggests that the profile of the homeless population in the Netherlands has changed substantially over recent years. This might be due (in part) to the influence of local and national policy by which the ‘traditional’ homeless populations, including the chronically homeless, have largely been successfully taken off the streets in recent decades (Barendregt and van de Mheen 2009; Tuynman and Planije 2014). Also, considerable efforts have been made to improve the situation of homeless people during the study period (Dutch Government and Four Major Cities 2011). These factors might account for the improvements in the social exclusion indicators among the participants in the present study. However, despite these positive results, between 2009 and 2012 the estimated size of the homeless population in the Netherlands increased, which was largely due to the financial crisis (Coumans et al. 2015). It is estimated that in 2015 a total of 31,000 people were homeless in the Netherlands (Statistics Netherlands 2016).

### Strengths and Limitations

The present study has a number of strengths. Longitudinal data were available for a relatively large cohort of homeless people with a very high response rate at follow-up (73.7%). Our investigation of social exclusion indicators among homeless people provided broad insight into their disadvantaged situation. Also, using longitudinal data and investigating indicators of social exclusion placed the situation of homeless people in a broader perspective. Finally, as homeless people are often the least likely to be included in common measures of social exclusion (Popay et al. 2008), we offer insight into indicators of social exclusion of this very vulnerable group.

Some limitations also need to be addressed. One of the main challenges when studying social exclusion among populations is the selection of appropriate indicators for social exclusion (Coumans and Schmeets 2015). We selected indicators proven to be relevant when studying homeless people. Although our conceptualization of social exclusion was more appropriate for homeless people than the commonly used indicators for the general population, we may have used too narrowly defined indicators of social exclusion. Moreover, we did not use the same questionnaire as used among the general population in the study of Jehoel-Gijsbers et al. (2009), and did not ask the participants directly whether or not they felt socially excluded, which might have provided additional information apart from the more conceptual measures. The indicators we selected for this study were related to the four-dimensional model of social exclusion (Jehoel-Gijsbers and Vrooman 2007). We explored whether these indicators represented the four-dimensional model of social exclusion developed for the general population (Jehoel-Gijsbers and Vrooman 2007) by means of a categorical principal components analysis. This analysis showed that some of the selected indicators matched the social exclusion dimensions, but the dimensions were not fully covered. Thus, the internal validity with regard to the measurement of the social exclusion dimensions might be compromised. Future studies are required on how best to measure social exclusion among homeless people and which indicators should be included.

Another issue is the higher loss to follow-up of participants who were younger, male, and had the lowest level of education. However, it is unknown whether and in which direction this selective loss to follow-up may have biased our findings as we lack information on the change variables of these non-respondents which we used in the analyses.

An issue related to the construction of the variables is the dichotomization of debts and psychological distress. Debts were dichotomized because they showed skewed distributions and multiple outliers and this dichotomization could decrease statistical power. However, an advantage of dichotomization is that it encourages a ‘risk factor’ approach, which helps in targeting intervention efforts (Farrington and Loeber 2000). The dichotomization of psychological distress was based on age and gender-adjusted norm scores of psychological distress of a Dutch community sample, which helped our understanding of the results and the magnitude of psychological distress in our participants. To check the possible impact of this dichotomization, we additionally analysed the data by means of a linear regression with psychological distress as a continuous variable. This analysis revealed the same significant variables as the analyses with psychological distress as a categorical variable.

A final methodological issue concerns the external validity of the results. We studied a subgroup of the total population of homeless people in the Netherlands, i.e. those who reported themselves at a central access point for social relief in 2011 in one of the four major Dutch cities and were accepted for an individual program plan. As stated before, it is obligatory for every homeless person to report at a central access point for social relief in order to gain access to social relief facilities (e.g. a night shelter). Therefore, the vast majority of the homeless population is covered when using this selection criterion. Subgroups of homeless people not included in this study were undocumented homeless people and homeless people who do not make use of social relief facilities. These latter groups may show different patterns regarding social exclusion indicators and psychological distress.

Several questions concerning the concept of social exclusion and the application of the concept for homeless people remain. For example, there is no consensus on which dimensions are relevant, which (if any) are the most important, and whether being socially excluded is an objective state or a subjectively felt experience (Morgan et al. 2007).

## Conclusion

Although homelessness is inherently associated with social exclusion, homeless individuals are rarely included in conventional studies on social exclusion. This study focused on changes in indicators of social exclusion among homeless people over a 2.5-year period, and associations between changes in indicators of social exclusion and changes in psychological distress. Improvements were found in various indicators of social exclusion, whereas financial debts showed no significant improvement. Changes in unmet care needs, health insurance, social support from family and relatedness to others were related to changes in psychological distress. By using a four-dimensional concept of social exclusion, this study shed light on the concept of social exclusion among homeless people.
